# The Selective Histone Deacetylase Inhibitor MI192 Enhances the Osteogenic Differentiation Efficacy of Human Dental Pulp Stromal Cells

**DOI:** 10.3390/ijms22105224

**Published:** 2021-05-14

**Authors:** Kenny Man, Liam Lawlor, Lin-Hua Jiang, Xuebin B. Yang

**Affiliations:** 1 Biomaterials and Tissue Engineering Group, School of Dentistry, University of Leeds, Leeds LS9 7TF, UK; k.l.man@bham.ac.uk (K.M.); liam_lawlor@hotmail.com (L.L.); 2 School of Chemical Engineering, University of Birmingham, Edgbaston, Birmingham B15 2TT, UK; 3 School of Biomedical Sciences, University of Leeds, Leeds LS2 JT, UK; l.h.jiang@leeds.ac.uk

**Keywords:** histone deacetylase, HDAC inhibitor, epigenetics, MI192, bone tissue engineering, human dental pulp stromal cells

## Abstract

The use of human dental pulp stromal cells (hDPSCs) has gained increasing attention as an alternative stem cell source for bone tissue engineering. The modification of the cells’ epigenetics has been found to play an important role in regulating differentiation, with the inhibition of histone deacetylases 3 (HDAC3) being linked to increased osteogenic differentiation. This study aimed to induce epigenetic reprogramming using the HDAC2 and 3 selective inhibitor, MI192 to promote hDPSCs osteogenic capacity for bone regeneration. MI192 treatment caused a time–dose-dependent change in hDPSC morphology and reduction in viability. Additionally, MI192 successfully augmented hDPSC epigenetic functionality, which resulted in increased histone acetylation and cell cycle arrest at the G_2_/M phase. MI192 pre-treatment exhibited a dose-dependent effect on hDPSCs alkaline phosphatase activity. Quantitative PCR and In-Cell Western further demonstrated that MI192 pre-treatment significantly upregulated hDPSCs osteoblast-related gene and protein expression (alkaline phosphatase, bone morphogenic protein 2, type I collagen and osteocalcin) during osteogenic differentiation. Importantly, MI192 pre-treatment significantly increased hDPSCs extracellular matrix collagen production and mineralisation. As such, for the first time, our findings show that epigenetic reprogramming with the HDAC2 and 3 selective inhibitor MI192 accelerates the osteogenic differentiation of hDPSCs, demonstrating the considerable utility of this MSCs engineering approach for bone augmentation strategies.

## 1. Introduction

An increasing number of people suffer from bone damage or loss caused by traumatic injury, tumour resection or common age-associated diseases such as osteoporosis [1], and this is expected to rise in the future, due to increasing life expectancy. Although bone has the intrinsic ability for self-repair and regeneration, bone damage reaching a critical size potentially results in non-union or permanent defects requiring surgical intervention [2,3]. The annual cost of musculoskeletal disorders to the UK Health Service is £4.76 billion [4]. Thus, the ability to generate new bone is still a major clinical need which creates an enormous social and economic burden. Current clinical therapies such as autografts are seen as the gold standard [5,6], however, these procedures are highly invasive, with limited tissue availability and inducing donor site morbidity [7,8]. Consequently, extensive research has been conducted in the tissue engineering field to meet the rising demand for clinically relevant bone tissue. The key to tissue engineering approaches is to effectively control the lineage-specific differentiation of mesenchymal stromal cells (MSCs) [9,10]. Bone marrow derived MSCs have been extensively utilised for bone augmentation strategies, however, there has been limited clinical success due to issue including low procurement yield, extensive in vitro expansion required and their inherent heterogeneity [11,12]. Human dental pulp stromal cells (hDPSCs) have garnered increasing interest as an alternative MSC source for use in bone tissue engineering due to the ease of tissue extraction, their high proliferation rate and multipotency [13,14]. 

In recent years, several cell engineering approaches have been investigated to enhance the efficacy of MSCs for bone augmentation strategies. Methods such as gene therapy have provided much excitement within the field [15], however, due to the low process efficacy, cost-intensiveness and the potential risk of virally induced tumorigenicity [16], alternative methods of controlling MSCs osteogenesis have been explored. It has become increasingly apparent that epigenetics plays a significant role in regulating cell fate. Specifically, researchers have demonstrated that post-translational modifications such as the process of acetylation results in alterations in the chromatin structure. This ultimately affects the transcriptional activity of the cell via two groups of histone-modifying proteins, histone acetyltransferase (HAT) and histone deacetylase (HDAC) [17]. HAT is responsible for the acetylation of histones, which results in the opening of the chromatin structure, increasing the cells’ transcriptional activity, while histone deacetylation by HDAC leads to transcriptional repression via condensation of the chromatin. Such modifications have been showed to affect MSC functions such as multipotency and differentiation potential [17,18]. 

**Figure 1:**
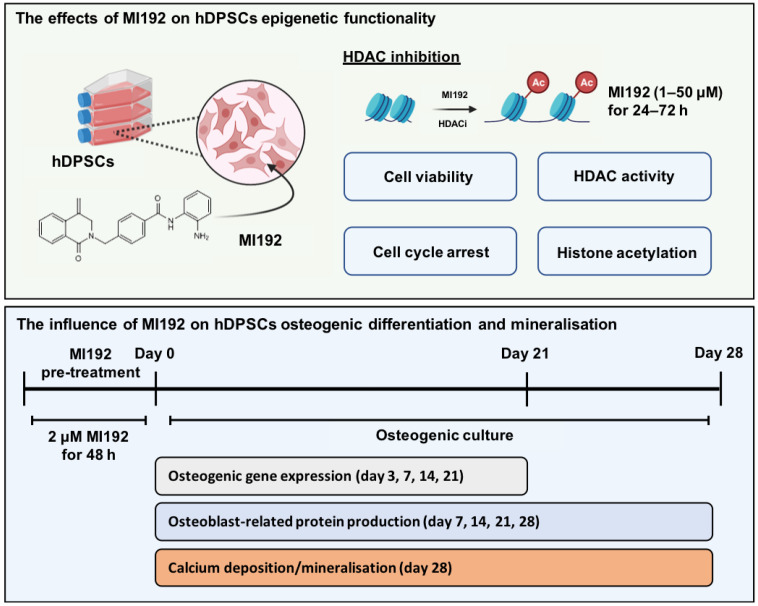
Experimental outline investigating the effects of altering hDPSCs epigenetic functionality with MI192 on their osteogenic capacity.

To date, HDAC inhibitors (HDACi) such as Romidepsin and Vorinostat (SAHA) have been approved by the Food and Drug Administration (FDA) for the treatment of cutaneous T-cell lymphoma [19]. HDACis can deregulate the epigenetic process and induce hyperacetylation resulting in enhancing gene transcription, promoting MSC osteogenic differentiation [20,21,22,23]. Although these approaches have shown promise, the use of non-selective panHDACis is associated with limitations such as unwanted side effects and reduced differentiation efficacy by targeting a broad range of HDAC isoforms [24]. Hence, there is a growing precedence to move towards using a more selective approach. Studies have reported the role of HDAC3 in regulating osteogenic differentiation by acting as a co-repressor of RUNX2, a transcription factor responsible for modulating osteocalcin production, known to be essential for bone formation [25]. MI192 is a novel benzamide derivative and a selective HDAC2 and 3 inhibitor, which has demonstrated its potential use in the treatment of leukaemia and rheumatoid arthritis [26,27]. Moreover, we have previously demonstrated the increased efficacy of MI192 on promoting the osteogenic differentiation of adipose-derived MSCs (ADSCs) when compared to the use of the panHDACi Trichostatin A (TSA) [28]. Therefore, in this study we investigated the effects of augmenting hDPSCs epigenetic functionality with the HDAC2 and 3 selective inhibitor MI192 to enhance its efficacy for bone tissue engineering (Figure 1).

## 2. Results

### 2.1. MI192 Affects hDPSCs Morphology and Viability

**Figure 2:**
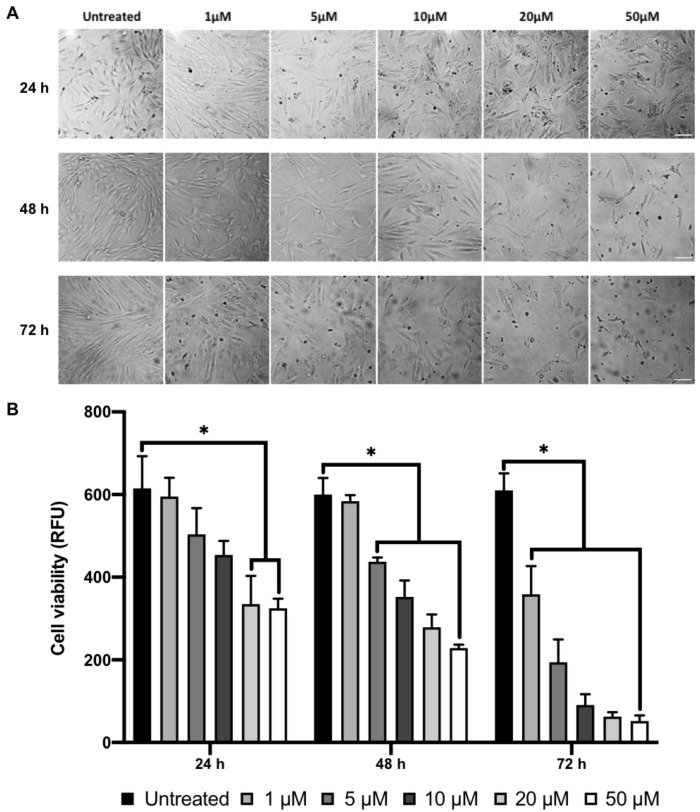
Effects of MI192 on hDPSCs morphology and viability. (**A**) hDPSCs morphology. Scale bar = 100 μm. (**B**) Metabolic activity of hDPSCs, quantified with the AlamarBlue assay. Data are expressed as mean ± SD (*n* = 3). * *p* ≤ 0.05.

The hDPSCs were treated with various concentrations of MI192 and incubated for up to 72 h. Following 24 h of culture, untreated hDPSCs exhibited a typical fibroblast-like morphology (Figure 2A). MI192 at low concentrations (1 and 5 μM) induced a slight elongation in the cell morphology and at higher doses (>10 μM), there was a concentration-dependant reduction in cell density. Treatment with MI192 at high concentrations (>10 μM) for 48 and 72 h resulted in a time–dose-dependent decrease in cell density, with an increasing number of floating cells observed. AlamarBlue assay showed that MI192 caused a time–dose-dependent reduction in hDPSCs metabolic activity when compared to the untreated cells (Figure 2B). Treatment with MI192 at concentrations of ≥20 μM for 24 h, ≥5 μM for 48 h and ≥1 μM for 72 h significantly reduced hDPSCs metabolic activity compared to that in the untreated cells (*p* ≤ 0.05).

### 2.2. MI192 Altered hDPSCs HDAC Activity and Histone Acetylation

**Figure 3:**
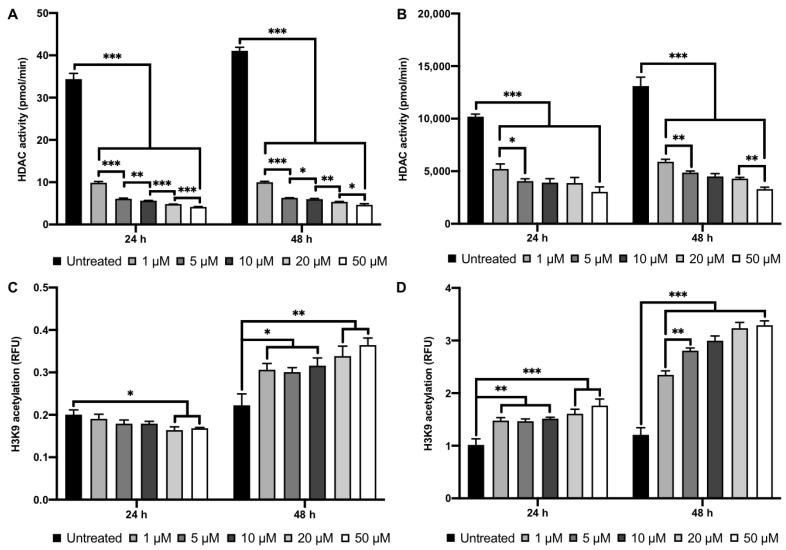
The influence of MI192 on hDPSCs epigenetic functionality. HDAC activity levels (**A**) immediately post MI192 treatment and (**B**) 1 week post MI192 treatment. H3K9 histone acetylation levels (**C**) immediately post MI192 treatment and (**D**) 1 week post MI192 treatment. Data are expressed as mean ± SD (*n* = 3). * *p* ≤ 0.05, ** *p* ≤ 0.01 and *** *p* ≤ 0.001.

Treatment with 1–50 µM MI192 for 24 and 48 h led to a significant reduction in hDPSCs HDAC activity (≥3.4-fold and 4.1-fold, respectively) compared to that of the untreated cells in a dose-dependent manner (*p* ≤ 0.001) (Figure 3A). Moreover, 1 week post MI192 treatment for 24 and 48 h, the HDACi treated cells exhibited a similar significant reduction in HDAC activity (≥1.95-fold and 2.25-fold) compared to the untreated cells in a dose-dependent manner (*p* ≤ 0.001) (Figure 3B). MI192 treatment for 24 h caused a dose-dependent reduction in the histone acetylation levels, where MI192 at concentrations greater than 20 μM significantly decreased the acetylation levels (≥1.2-fold) compared to that in the untreated cells (*p* ≤ 0.05) (Figure 3C). However, MI192 treatment for 48 h led to a significant and dose-dependent increase in the histone acetylation levels (≥1.4-fold) compared to that in the untreated cells (≤10 µM, *p* ≤ 0.05) (≥20 µM, *p* ≤ 0.01). One week post MI192 treatment for 24 and 48 h, the HDACi treated cells displayed a significant dose-dependent increase in histone acetylation levels (≥1.45-fold and 1.95-fold) when compared to the untreated control (*p* ≤ 0.01–0.001) (Figure 3D).

### 2.3. MI192 Halts hDPSCs Cell Cycle Progression

**Figure 4:**
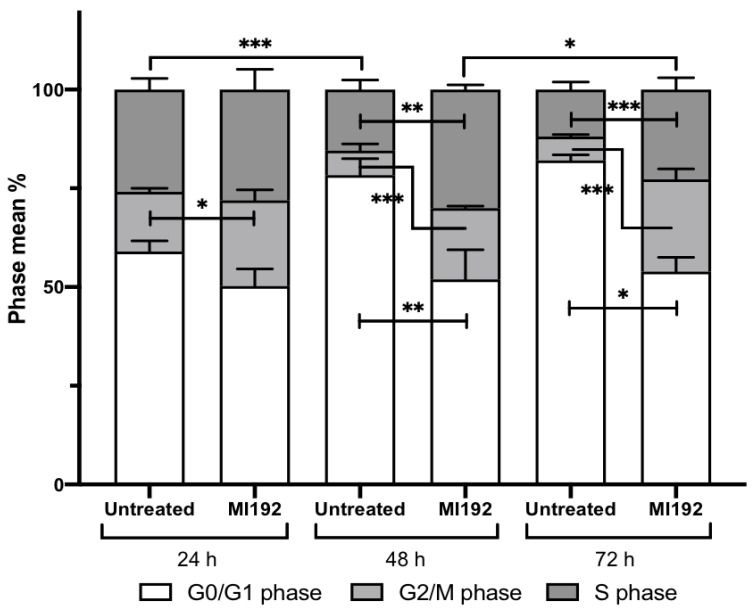
Effects of MI192 treatment on hDPSCs cell cycle progression. Data are expressed as mean ± SD (*n* = 3). The significance levels shown are the test group compared to the basal control for that time point. * *p* ≤ 0.05, ** *p* ≤ 0.01 and *** *p* ≤ 0.001.

The effects of MI192 treatment (2 µM) on hDPSCs cell cycle progression was assessed following treatment for 24, 48 and 72 h using flow cytometry. The percentage of cells distributed within the different phases of the cell cycle is shown in Figure 4.

#### 2.3.1. G_0_/G_1_ Phase

At 24 h, there was a non-significant reduction in the percentage of MI192 treated cells in the G_0_/G_1_ phase (50%) compared to the untreated group (59%) (*p* > 0.05). However, at 48 h, the number of cells significantly decreased in the MI192 treatment group (52%) compared to the control group (78%) (*p* ≤ 0.01). A similar pattern was observed at 72 h, with MI192 treatment (82%) significantly reducing the percentage of cells compared to the control group (52%) (*p* ≤ 0.05).

#### 2.3.2. G_2_/M Phase

The proportion of cells in the G_2_/M phase within the MI192 treatment group (22%) was significantly increased compared to the control group (15%) at 24 h (*p* ≤ 0.05). At 48 h, the MI192 treated cells (19%) continued to exhibit a significantly enhanced percentage compared to the control group (6%) (*p* ≤ 0.001), and at 72 h, a similar trend was observed where MI192 treatment (23%) substantially increased the percentage of cells in the G_2_/M phase compared to the control group (6%) (*p* ≤ 0.001).

#### 2.3.3. S Phase

After 24 h, MI192 treatment (28%) induced a non-significant increase in the percentage of cells in the S phase compared to the control group (26%) (*p* > 0.05). At 48 h, a significant increase was observed in the MI192 group (29%) compared to the control group (15%) (*p* ≤ 0.01). At 72 h, the MI192-treated cells (23%) maintained a significant enhancement compared to the untreated cells (12%) (*p* ≤ 0.001).

### 2.4. MI192 Exhibited a Dose-Dependent Effect on hDPSCs Alkaline Phosphtase (ALP) Activity

**Figure 5:**
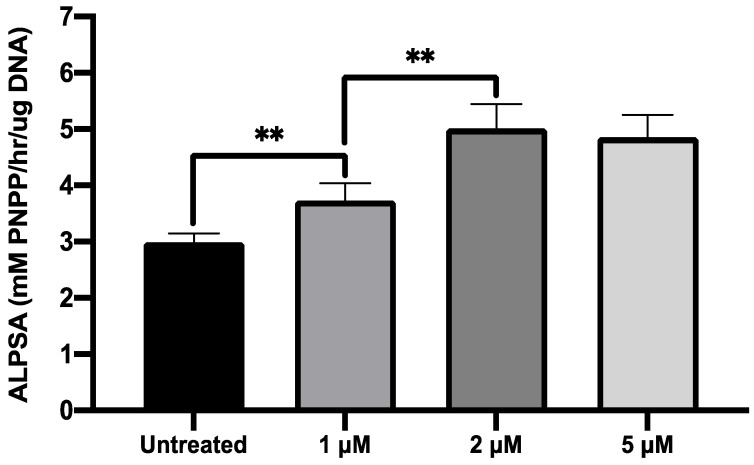
Effect of MI192 pre-treatment on hDPSCs ALPSA. Cells were pre-treated with/without MI192 for 48 h before culture in osteogenic conditions for 14 days. Data are expressed as mean ± SD (*n* = 3). ** *p* ≤ 0.01.

The effects of MI192 treatment on hDPSCs osteogenic differentiation was initially evaluated by quantifying ALP specific activity (ALPSA). Cells were treated with 1, 2, 5 µM MI192 for 48 h before osteoinductive culture for 14 days. Pre-treatment with all MI192 concentrations significantly enhanced ALPSA (≥1.29-fold) compared to the untreated cells (*p* ≤ 0.01) (Figure 5). Treatment with 2 and 5 µM MI192 elicited a 1.68 and 1.62-fold increase in ALPSA levels compared to the untreated group (*p* ≤ 0.01), however, no statistical difference was observed between the groups (*p* > 0.05). Therefore, a pre-treatment regimen of 2 μM MI192 for 48 h prior to osteoinductive culture was used for the rest of this study. 

### 2.5. MI192 Promoted the Osteogenic Gene and Protein Expression in hDPSCs

**Figure 6:**
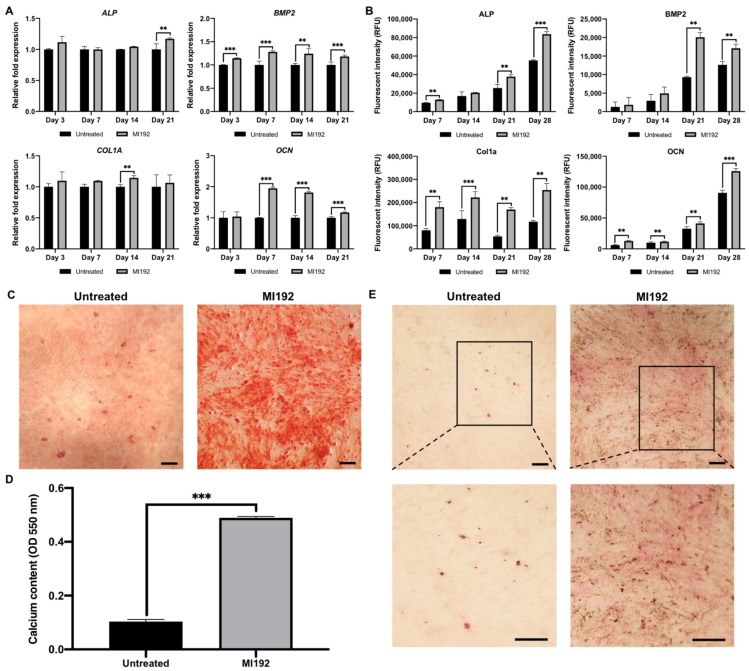
The effects of MI192 pre-treatment (2 μM MI192 for 48 h) on hDPSCs osteogenic differentiation and mineralisation. Osteoblast-related (**A**) gene expression, (**B**) protein production, (**C**,**D**) calcium deposition (scale bar = 100 µm)) (day 28) and (**E**) mineral nodule formation (day 28) (scale bar = 50 µm). Data are expressed as mean ± SD (*n* = 3). ** *p* ≤ 0.01 and *** *p* ≤ 0.001.

The effects of MI192 pre-treatment on hDPSCs osteoblast-related gene and protein expression were examined by RT-qPCR and ICW, respectively (Figure 6). The MI192 pre-treated groups exhibited a slight non-significant increase in *ALP* mRNA expression compared to that in the untreated cells at day 3, 7 and 14 (*p* > 0.05) (Figure 6A). However, on day 21, *ALP* mRNA levels were significantly enhanced in the MI192 pre-treated cells compared to that of the untreated group (*p* ≤ 0.01). The ALP protein expression was significantly and consistently upregulated in the MI192 pre-treated cells on days 7, 21 and 28 (1.35, 1.48 and 1.51-fold, respectively) compared to that in the untreated group (*p* ≤ 0.01, days 7 and 21) (*p* ≤ 0.001, day 28) (Figure 6B). The MI192 pre-treated cells exhibited a significant increase in bone morphogenic protein 2 (*BMP2)* mRNA expression from day 3 through day 21 (1.15, 1.28, 1.23 and 1.18-fold, respectively) (*p* ≤ 0.01, day 14) (*p* ≤ 0.001, day 3, 7 and 21) compared to the untreated cells (Figure 6A). Similarly, the BMP2 protein expression was significantly upregulated in the MI192 pre-treated group compared to that in the untreated cells at days 21 and 28 (2.16 and 1.35-fold, respectively) (*p* ≤ 0.01) (Figure 6B). The MI192 pre-treated group exhibited a non-significant increase in type I collagen (*COL1A)* mRNA expression levels compared to the untreated group at days 3, 7 and 21 (*p* > 0.05) (Figure 6A). However, a significant enhancement in the *COL1A* mRNA expression levels was observed in the MI192 pre-treated group at day 14 (1.14-fold) compared to that in the untreated cells (*p* ≤ 0.01). The Col1a protein expression was significantly upregulated in the MI192 pre-treated cells on days 7, 14, 21 and 28 (2.24, 1.71, 3.15 and 2.17-fold, respectively) compared to the untreated group (*p* ≤ 0.01, days 7, 21 and 28) (*p* ≤ 0.001, day 14) (Figure 6B). Osteocalcin (*OCN)* mRNA expression levels in the MI192 pre-treated cells were significantly enhanced by 1.95-fold on day 7 (*p* ≤ 0.001), 1.81-fold on day 14 (*p* ≤ 0.001) and 1.17-fold on day 21 (*p* ≤ 0.001) compared to the untreated group (Figure 6A). The OCN protein expression level was significantly elevated in the MI192 pre-treated group (2.12, 1.15, 1.26 and 1.4-fold, respectively) compared to that in the untreated cells on days 7, 14, 21 and 28 (*p* ≤ 0.01, days 7, 14 and 21) (*p* ≤ 0.001, day 18) (Figure 6B).

### 2.6. MI192 Enhanced hDPSCs Extracellular Matrix Mineralisation

The effects of MI192 pre-treatment on hDPSCs extracellular matrix calcium deposition was identified via Alizarin red staining. MI192 substantially enhanced hDPSCs alizarin red staining for calcium deposition compared to that in the untreated cells (Figure 6C). Following semi-quantitative analysis, it was confirmed that the MI192 pre-treated group exhibited a significant increase in calcium deposition (4.75-fold) compared to that in the untreated cells (Figure 6D) (*p* ≤ 0.001). Von Kossa analysis was performed to assess the formation of mineral nodules in the extracellular matrix. A substantial increase in mineral nodules (black staining) was observed within the MI192 pre-treated cells compared to that in the control group (Figure 6E). Additionally, enhanced Van Gieson’s staining for collagen deposition (pink staining) was observed in the MI192 pre-treated group, particularly situated in close proximity to Von Kossa positive mineral nodules.

## 3. Discussion

Epigenetic approaches for tissue engineering have gained increasing attention due to their ability to modulate cellular functions without altering the DNA sequence. An increasing number of reports have demonstrated the feasibility of using HDACis to enhance the osteogenic potential of MSCs [29,30]. However, the majority of these studies have utilised non-selective panHDACis, which are associated with reduced efficacy and potential side effects [24]. The HDAC3 isoform has been shown to be linked to osteogenic differentiation due to its repression of the osteogenic transcription factor RUNX2 [31]. MI192 enhanced selectivity for HDAC2 and 3 isoforms has increased its efficacy for different clinical applications [26,27], hence, the use of this selective approach may promote the clinical utility of MSC-based therapies for bone tissue engineering applications.

HDPSCs have garnered growing interest for use in bone tissue engineering, due to their ease of procurement, increased proliferation rate and osteogenic capacity [13,32]. Therefore, the combination of this stem cell source with HDACi treatment could further accelerate the fracture healing process which would have a tremendous impact in the clinical practice. Several studies have reported the ability of HDACis to initiate cell death, which is key to their utility in cancer therapeutics [30,33]. To enhance the safety and efficacy of HDACi-based therapeutics, a clearer understanding of the effects of these epigenetic regulators on normal cells [34], in particular on MSCs, is essential for clinical translation. The findings of this study showed that MI192 treatment caused a time–dose-dependent reduction in the viability of hDPSCs. Boissinot et al. (2012) showed a similar pattern with MI192 inducing a time–dose-dependent reduction in the viability of leukemic cell lines (HL60, Kasumi-1 and U937) [26]. These findings suggest that MI192 impact on cell viability is not only limited to cancer cell lines but also stem cells, an important consideration for the use in MSCs-based tissue engineering.

Several studies suggest that HDACis can arrest cells in various stages of the cell cycle [35,36]. Additionally, it has been reported that the HDAC2 and 3 isoforms are involved in regulating cell cycle progression [37,38]. In the present study, treatment with 2 μM MI192 caused a time-dependent reduction in the accumulation of cells in the G_0_/G_1_ phase but increased the percentage of cells in the S and G_2_/M phase. Jiang et al. (2014) described similar results, where adult neural stem cells with reduced expression of HDAC3 were unable to pass the G_2_/M phase, indicating the role of HDAC3 in controlling the cell cycle progression through this phase [38]. Treatment with 2 μM MI192 was shown to exhibit limited toxicity from the viability assessment, thus it is probable that the effects on cell cycle progression is associated with halting cellular proliferation, therefore priming cells for differentiation, and leading to cell synchronization rather than initiating cell death.

Since MSCs are highly receptive to epigenetic changes [39], the use of HDACis may provide a valuable approach to enhance the efficacy of MSCs-based therapies for bone tissue engineering applications. Therefore, it is important to elucidate the mechanism in which MI192 regulates hDPSCs epigenetic functionality. In this study, MI192 treatment significantly reduced the HDAC activity in a time–dose-dependent manner compared to untreated cells. This correlated with the effects of MI192 on HeLa and PC3 cells [26,40], where treatment with 10 μM MI192 for 24 h reduced HDAC activity by 49% compared to the untreated group. Our results showed a reduction of 83% in HDAC activity after 24 h treatment with 10 μM MI192, indicating MSCs may be more sensitive to MI192 compared to the transformed cells. Following 1-week post 24 and 48 h of MI192 treatment, the HDACi treated cells continued to exhibit a significant reduction in the HDAC activity when compared to the untreated cells (~49% reduction). The prolonged inhibitory effect of this compound is likely due to the slow binding kinetics of MI192, correlating with the observations reported by Boissinot et al. [26]. More importantly, our data showed that the HDAC inhibition induced by MI192 after 48 h resulted in a positive enhancement on histone H3K9 acetylation. The increased H3K9 acetylation represents a transcriptionally permissive chromatin structure which has been reported to enhance gene expression [41,42], therefore, the introduction of osteogenic growth factors to cells with a transcriptionally active/open chromatin structure will likely enhance growth factor induced differentiation efficacy within hDPSCs. Moreover, it has been reported that hyperacetylation promotes the transcriptional activity of Runx2. Jeon et al. (2006) showed that Runx2 hyperacetylation enhanced the activity and stability of this transcription factor via protection from smurf-1 mediate degradation [43]. Hence, it is likely the MI192 induced hyperacetylation within hDPSCs enhanced the transcriptional activity of Run2, however, this would require further investigation. Importantly, it was also confirmed that the prolonged HDAC inhibition observed after 1 week of culture resulted in increased histone acetylation levels within the MI192 pre-treated cells. This indicates the slow on/off binding kinetics of MI192 [26], potentiating the effects of the altered epigenome on hDPSCs osteogenic differentiation. Taken together, these results confirm that MI192 is an effective epigenetic regulator within hDPSCs, and the augmented epigenome induced by HDACi treatment potentially indicates the increased differentiation capacity of these cells.

HDACis have been shown to alter the expression of osteoblast-related markers in MSCs by regulating their epigenetic functionality [20,30,44]. In this study, the effects of MI192 pre-treatment on the expression of osteoblast-related genes and proteins were investigated. Our finding showed that MI192 significantly upregulated hDPSCs BMP2 expression levels throughout osteogenic culture. Phimphilai et al. (2006) proposed that BMP signalling is an essential component to RUNX2-dependent osteogenesis [45], and Hu et al. (2013) confirmed this in ADSCs with the addition of BMP antagonist, Noggin, resulting in a decrease in the RUNX2 mRNA expression level [46]. Therefore, the upregulation in BMP2 expression levels, induced by MI192 treatment, indicates the HDACis role in stimulating hDPSCs early phase osteogenic differentiation. Studies have also reported that BMP2 levels increases steadily throughout hDPSC osteogenesis [30,47], correlating with the mRNA expression profiles in this study. MI192 pre-treatment also upregulated the gene and protein expression of ALP, which was confirmed by the corresponding functional ALPSA assay. The mRNA and protein expression levels of COL1A, known to be a key component of the bone extracellular matrix [48], was significantly upregulated in the MI192 pre-treated hDPSCs throughout osteogenic culture, indicating the role of MI192 in accelerating the maturation of the extracellular matrix. Additionally, MI192 promoted the OCN levels when compared to untreated cells, indicating that MI192 pre-treatment is capable of promoting hDPSC osteogenic maturation. Piano et al. (2014) reported that valproic acid (VPA) pre-treated hDPSCs exhibited significantly reduced OCN mRNA and protein expression, indicating that VPA inhibited osteogenic maturation. Additionally, this group demonstrated that silencing HDAC2 inhibited the expression of OCN in Saos-2M osteosarcoma cell line, suggesting the importance of HDAC2 in regulating the OCN expression [30,49]. In the present study, we did not observe this downregulation in the OCN expression following MI192 pre-treatment, likely due to the selective inhibition of MI192 on HDAC3, which VPA does not possess. Schroeder et al. (2004) reported the importance of the RUNX2-HDAC3 complex in regulating the mRNA and protein expression of OCN [25]. Due to MI192 selective inhibition of HDAC3, this probably alleviates HDAC3 repression of RUNX2, leading to increased activity and stability of this transcription factor [42], although this would require further investigation. Taken together, these results show that hyperacetylation induced by MI192 pre-treatment increase the expression of hDPSCs osteoblast-related markers, consistent with several studies in the literature [50,51,52]. Although enhanced expression levels of osteoblast-related genes and proteins was observed in the MI192 pre-treated hDPSCs, it is likely HDACi-induced epigenetic modification altered the processing of transcribed mRNA into function proteins through altered RNA splicing/microRNA expression [53,54], which would also be of interest to be elucidated in future studies. Additionally, it is important to note that the elevated osteogenic capacity induced by MI192 pre-treatment is likely due to a combination of enhanced accessibility to genes of interest via increased chromatin transcriptional activity and transcription factor activation via hyperacetylation and HDAC isoform inhibition.

The effects of MI192 pre-treatment on calcium deposition and mineralisation were assessed with Alizarin red and Von Kossa staining, respectively [55]. MI192 substantially enhanced extracellular matrix collagen deposition and mineralisation compared to the untreated hDPSCs, correlating with the increased mineralisation observed in MI192 pre-treated ADSCs [28]. These findings clearly indicate that MI192 effects in stimulating hDPSCs efficacy for bone formation is not limited to enhancing osteogenic differentiation but also the mineralisation of its extracellular matrix, consistent with observations in the literature [32,56]. Moreover, the enhanced collagen production and calcium deposition observed in this study indicate the efficacy of MI192 pre-treatment in promoting hDPSCs mineralisation, likely due to the slow-binding kinetics of this HDACi [26]. The effectiveness of this pre-treatment strategies enhances the utility of MI192 in cell-based therapies for bone augmentation strategies.

In the present study, we examined the influence of altering hDPSCs epigenetic functionality with the HDAC2 and 3 selective inhibitor, MI192, in promoting their efficacy for bone augmentation strategies. We have shown that MI192 is an effective epigenetic regulator within hDPSCs, augmenting key cellular processes including metabolic activity and cell cycle progression through HDACi induced hyperacetylation. Importantly, we have demonstrated that utilising a pre-treatment regimen, MI192 accelerated the osteogenic differentiation and mineralisation rate of hDPSCs, enhancing its bone formation capacity. Future studies would be required to further examine the influence of MI192 treatment on stimulating bone regeneration in vivo.

## 4. Materials and Methods

### 4.1. Cell Culture

HDPSCs were isolated from sound third molars obtained with patients’ consent through the Leeds Dental Institute Research Tissue Bank (REC 07/H1306/93), with ethical approval (180,615/km/173) [13]. HDPSCs were isolated using the collagenase digestion method previously described by Tomlinson et al. (2015) [57]. Cells were subsequently cultured in a basal medium consisting of alpha modified minimum essential medium (α-MEM, Lonza, Manchester, UK) supplemented with 10% fetal calf serum (FCS, Lonza, Manchester, UK), 2 mM L-glutamine and a mix of 100 units/mL penicillin with 100 μg/mL streptomycin at 37 °C in 5% CO_2_. Cells were passaged when reaching approximately 80% confluence, and hDPSCs of up to passage 4 were used for all experiments.

### 4.2. Cell Viability Assay and Morphological Assessment

For cell viability assays, hDPSCs were plated at 1 × 10^4^ cells per well within a 96-well plate in basal medium and incubated for 24 h. Media was then replaced with fresh basal media supplemented with MI192 at a range of concentrations (1, 5, 10, 20, 50 μM) and incubated for 24, 48 and 72 h. Basal medium alone was used as the control. Then, 20 µL of AlamarBlue reagent (Thermo Scientific, Paisley, UK) was added to each well and incubated for 4 h at 37 °C. Fluorescence readings were acquired using a Varioskan Flash Multimode Microplate Reader (Thermo Scientific, Paisley UK) at an excitation wavelength of 540 nm and emission wavelength at 590 nm. For cell morphological assessment, hDPSCs were seeded at 1 × 10^5^ cells per well in a 24-well plate and incubated for 24 h, after which the same treatment regime was utilised where the cell morphology was visualised by inverted microscopy (ZEISS, AX-100).

### 4.3. DNA Quantification

DNA content was determined by Quant-iT PicoGreen dsDNA Assay Kit (Invitrogen, Life Technologies, Paisley, UK) according to the manufacturer’s instructions. Briefly, cells were washed twice with phosphate-buffered saline (PBS, Lonza, Manchester, UK) and lysed following three freeze–thaw cycles in 0.1% Triton X-100 (Sigma-Aldrich, Gillingham, UK) in PBS. Then, 10 μL of cell lysate was added to 90 μL of TE buffer (10 mM Tris-HCl, 1 mM EDTA) into a 96-well plate (Corning, Deeside, UK). Next, 100 μL of PicoGreen reagent was added to all samples and then incubated in the dark for 5 min. The fluorescence was measured in a Varioskan Flash Multimode Microplate Reader at 480 nm excitation and 520 nm emission.

### 4.4. HDAC Activity

Cells were cultured in two 96-well plates (1 × 10^4^ cells per well) in basal medium containing MI192 at a range of concentrations (1, 5, 10, 20, 50 μM). Basal medium alone was used as the control. At 24 and 48 h, the HDAC activity in the cells was measured either measured immediately post treatment or following 1 week in basal culture using an InSitu HDAC Activity Fluorometric Assay Kit (Cambridge BioSciences, Cambridge, UK) according to the manufacturer’s instructions. Briefly, incubation media was replaced with 100 μL of reaction mix containing HDAC substrate and incubated for 3 h at 37 °C. Following which, 100 μL of lysine developer was added to the plate and incubated for a further 30 min at 37 °C. The fluorescence was measured in a Varioskan Flash Multimode Microplate Reader at an excitation wavelength of 368 nm and emission wavelength at 442 nm. HDAC activity was normalised with DNA content. 

### 4.5. Histone Acetylation

Detection of H3K9 histone acetylation was assessed using the EpiQuik In Situ Histone H3-K9 Acetylation Assay Kit (Epigentek, Farmingdale, NY, USA). Cells were cultured in basal medium containing different concentration of MI192 (1, 5, 10, 20, 50 μM) in a 96-well plate. Basal medium alone was used as the control. After treatment for 24 and 48 h, histone acetylation was performed either immediately post treatment or following 1 week in basal culture according to the manufacturer’s instructions. The absorbance was read on a Varioskan Flash Multimode Microplate Reader at 450 nm within 10 min. Acetylation was normalised to DNA content. 

### 4.6. Cell Cycle Analysis

Cells were plated in 6-well plates at 1 × 10^5^ cells per well and cultured in basal medium for 48 h. The medium in the test group was replaced with basal medium containing 2 μM MI192 and basal medium alone was used as the control. At 24, 48 and 72 h, cells were trypsinised, re-suspended with 500 μL of ice-cold 70% (*v*/*v*) ethanol/PBS and stored in −80 °C for assessment. For staining, the cell suspension was centrifuged at 800 rpm for 5 min, and the pellet was washed with 500 μL 4 °C FACS buffer: PBS with the addition of 0.1% (*w*/*v*) bovine serum albumin (Sigma-Aldrich, Gillingham, UK) and 0.1% TWEEN-20 (Sigma-Aldrich, Gillingham, UK). The cell pellet was re-suspended with 500 μL of freshly made staining solution (20 μg/mL propidium iodide (Sigma-Aldrich, Gillingham, UK) and 200 μg Ribonuclease A (Sigma-Aldrich, Gillingham, UK)) and incubated for 20 min at room temperature. Cells were then placed on ice for assessment with Attune NxT Flow Cytometer (Life Technologies, Paisley, UK). Subsequent cell cycle analysis was undertaken using ModFit LT software (LT v3, Verity).

### 4.7. MI192 Pre-Treatment and Osteogenic Induction 

HDPSCs were cultured in 24-well plates (4 × 10^4^ cells per well) in basal medium for 24 h. The media was replaced with basal medium containing 2 μM MI192 for 48 h. Basal medium alone was used as the control. Following the pre-treatment period, cells were washed with basal medium and incubated in osteogenic medium, which consisted of basal medium supplemented with 50 µM L-ascorbic acid 2-phosphate sesquimagnesium salt hydrate (Sigma-Aldrich, Gillingham, UK), 10 mM β-glycerol phosphate (Sigma-Aldrich, Gillingham, UK) and 100 nM dexamethasone (Sigma-Aldrich, Gillingham, UK). The medium was changed every 3 days.

### 4.8. Alkaline Phosphatase Specific Activity (ALPSA) Assay

ALPSA was determined using the 4-nitrophenyl colourimetric phosphate liquid system assay (pNPP, Sigma-Aldrich, Gillingham, UK). Briefly, following pre-treatment with/without 1, 2 and 5 µM MI192, hDPSCs were cultured in osteogenic medium for two weeks, and lysed with 0.1% Triton X-100 in PBS. Then, 90 μL of pNPP was added to 10 μL of cell lysate in a 96-well plate (Corning, Deeside, UK) for 30 min at 37 °C in the dark. Next, 100 μL of 1 M NaOH was added to each well to stop the reaction, and the absorbance was read at 405 nm on a Varioskan Flash Multimode Microplate Reader (Thermo Scientific, Paisley, UK). Alkaline phosphatase activity was normalised to total DNA content. 

### 4.9. Real-Time Reverse Transcription-Polymerase Chain Reaction (RT-qPCR)

Cells were pre-treated with/without 2 µM MI192 for 48 h followed by incubation in osteogenic medium for up to 28 days. At different time points (days 0, 3, 5, 7, 14, 21 and 28), cells were collected for RT-qPCR assay. The primers used in this study are listed in Table 1. The expression of osteoblast-related genes was assessed using RT-qPCR. RNA was extracted using the RNase mini kit (Qiagen, Manchester, UK) according to the manufacturer’s protocol. Then, 200 ng of RNA was transcribed to cDNA using the high capacity RNA to cDNA Kit (Applied Biosystems, Warrington, UK) following the supplier’s instructions. Following which, cDNA was amplified using TaqMan primers (Table 1) in a 20 µL reaction with a 96-well PCR plate (Starlab, Milton Keynes, UK). Amplification occurred within a LightCycler 480 real-time qPCR system (Roche, Welwyn Garden City, UK). For each sample, the cycle threshold (C_t_) value was acquired, and the comparative C_t_ method (2^−∆∆Ct^) was utilised to quantify the gene expression levels relative to the housekeeping gene.

### 4.10. In-Cell Western (ICW) Assay

The expression of osteoblast-related proteins was assessed using the ICW assay according to the manufacturer’s instructions. Briefly, cells were fixed in 10% neutral buffered formalin (NBF, Cellpath, Newtown, UK) for 20 minutes prior to be permeabilised with 0.1% Triton X-100 in PBS for 30 min and then blocked with LICOR Odyssey blocking buffer (Li-Cor Biosciences, Cambridge, UK) for 1.5 h at room temperature. The samples were incubated overnight at 4 °C with primary antibodies to ALP (1:300), COL1A (1:200), OCN (1:400) (Abcam, Cambridge, UK) and BMP2 (1:500) (Invitrogen, Life Technologies, Paisley, UK) in Odyssey buffer. Samples were washed in PBS containing 0.1% Tween 20 for five times and then incubated with the IRDye 800CW secondary antibody (1:800) and the CellTag 700 stain (1:500; Li-Cor Biosciences, Cambridge, UK) in the Odyssey blocking buffer for 1 h at room temperature [58]. The plates were read under the Odyssey SA Imaging System (Li-Cor Biosciences, Cambridge, UK) at both 700 and 800 nm. Relative fluorescent intensity (IRDye 800CW) was normalised with cell number (CellTag 700 stain). The data were quantified using the Image Studio analysis software (Li-Cor Biosciences: v5) [59].

### 4.11. Alizarin Red Staining and Quantification

Calcium-rich deposits were visualised using the Alizarin Red solution (Millipore, Watford, UK) for 15 min at room temperature, and observed under a Leica DMI6000 B inverted microscope. The resulting staining was dissolved in 10% cetylpyridinium chloride (Sigma-Aldrich, Gillingham, UK) for 1 h. Following this, the absorbance was read at 550 nm using the Varioskan Flash Multimode Microplate Reader.

### 4.12. Von Kossa Staining

Mineral nodules were identified using the Von Kossa Stain Kit (Atom Scientific, Cheshire, UK) according to the manufacturer’s instructions. Briefly, samples were incubated with 10% aqueous silver nitrate solution at room temperature in a UV lightbox for 10 min. This was followed by incubation with 5% sodium thiosulfate for 5 min and then incubated with Van Gieson’s solution for 5 min. The resultant staining was observed under a Leica DMI6000 B inverted microscope.

### 4.13. Data Analysis

For all data presented, experiments were repeated at least three times. All statistical analysis was undertaken with IBM SPSS software (IBM Analytics, version 21), using ANOVA and post-hoc Tukey’s test for comparisons of multiple groups. *p*-value equal to or lower than 0.05 was considered to be significant. For all graphs: * *p* ≤ 0.05, ** *p* ≤ 0.01 and *** *p* ≤ 0.001.

## 5. Conclusions

In conclusion, the findings of this study demonstrate that the selective HDAC2 and 3 inhibitor MI192 is capable of modifying the epigenome of hDPSCs, resulting in promoting its efficacy for bone formation. To the authors knowledge, this is the first study to promote the osteogenic capacity of hDPSCs via the use of a selectivity HDACi. Taken together, these findings demonstrated the considerable potential of using epige-netic regulation to enhance the efficacy of MSCs-based therapy for bone regeneration

## Figures and Tables

**Table 1:** Primers used for TaqMan gene expression assays.

Gene Symbol	Description	TaqMan Assay Identification
*GAPDH*	Glyceraldehyde-3-phosphate dehydrogenase	Hs99999905_m1
*BMP2*	Bone morphogenetic protein 2	Hs00154192_m1
*ALPL*	Alkaline phosphatase	Hs01029144_m1
*COL1A1*	Collagen, type I	Hs00164004_m1
*OCN/BGLAP*	Osteocalcin/PMF-bone gamma-carboxyglutamate (gla) protein	Hs00609452_g1
